# Genetic Architecture of Delayed Senescence, Biomass, and Grain Yield under Drought Stress in Cowpea

**DOI:** 10.1371/journal.pone.0070041

**Published:** 2013-07-30

**Authors:** Wellington Muchero, Philip A. Roberts, Ndeye N. Diop, Issa Drabo, Ndiaga Cisse, Timothy J. Close, Satoru Muranaka, Ousmane Boukar, Jeffrey D. Ehlers

**Affiliations:** 1 Department of Nematology, University of California Riverside, Riverside, California, United States of America; 2 Department of Botany & Plant Sciences, University of California Riverside, Riverside, California, United States of America; 3 Institut de l’environment et des Rechercheres Agricole, Kamboinse, Burkina Faso; 4 Senegalese Institute of Agricultural Research, Bambey, Senegal; 5 The International Institute of Tropical Agriculture, Ibadan, Nigeria; CIRAD, France

## Abstract

The stay-green phenomenon is a key plant trait with wide usage in managing crop production under limited water conditions. This trait enhances delayed senescence, biomass, and grain yield under drought stress. In this study we sought to identify QTLs in cowpea (*Vigna unguiculata*) consistent across experiments conducted in Burkina Faso, Nigeria, Senegal, and the United States of America under limited water conditions. A panel of 383 diverse cowpea accessions and a recombinant inbred line population (RIL) were SNP genotyped using an Illumina 1536 GoldenGate assay. Phenotypic data from thirteen experiments conducted across the four countries were used to identify SNP-trait associations based on linkage disequilibrium association mapping, with bi-parental QTL mapping as a complementary strategy. We identified seven loci, five of which exhibited evidence suggesting pleiotropic effects (stay-green) between delayed senescence, biomass, and grain yield. Further, we provide evidence suggesting the existence of positive pleiotropy in cowpea based on positively correlated mean phenotypic values (0.34< r <0.87) and allele effects (0.07< r <0.86) for delayed senescence and grain yield across three African environments. Three of the five putative stay-green QTLs, *Dro-1*, *3*, and *7* were identified in both RILs and diverse germplasm with resolutions of 3.2 cM or less for each of the three loci, suggesting that these may be valuable targets for marker-assisted breeding in cowpea. Also, the co-location of early vegetative delayed senescence with biomass and grain yield QTLs suggests the possibility of using delayed senescence at the seedling stage as a rapid screening tool for post-flowering drought tolerance in cowpea breeding. BLAST analysis using EST sequences harboring SNPs with the highest associations provided a genomic context for loci identified in this study in closely related common bean (*Phaseolus vulgaris*) and soybean (*Glycine max*) reference genomes.

## Introduction

Delayed senescence, grain yield, and biomass yield under drought stress rank among the most important traits targeted for improvement in crop plants [1]. However, these are genetically complex traits that are known to fall under the control of numerous genetic pathways [2]. As such, basic research targeted at isolating the genetic determinants is constrained by large numbers of small-effect quantitative trait loci (QTL) that, when treated as Mendelian factors, do not exert adequate impact at the phenotypic level to be detected easily. In practical crop breeding, complex breeding schemes have been required to capture sufficient QTL for economically viable selection gains in these traits.

The recent advent of cost-effective high-throughput genotyping technologies which enable construction of dense genetic maps and rapid genotyping of large germplasm collections has facilitated genome-level understanding of these traits and is helping to revolutionize marker-assisted breeding [3]. In general, accurate phenotyping for these traits has become the greatest constraint, typically requiring large amounts of time and material resources [1]. Primary productivity traits such as grain and biomass yield typically exhibit significant genotype by environment interactions (GxE), which necessitate extensive testing and breeding efforts targeted to specific production environments [4].

The stay-green trait, a phenomenon where QTLs for delayed senescence, biomass, and grain yield are co-located has been described in several crop species and is considered to be an important trait for improvement of yield under drought stress for cereals such as sorghum and maize [5]. Plants exhibiting the ‘stay-green’ phenotype are characterized by maintenance of green leaf area under drought stress. It is believed that the maintenance of green leaf area contributes to continued carbohydrate formation during drought and faster recovery following a rainfall event [5]. In cereal crops such as sorghum and maize, where the trait was first described, the stay-green phenotype manifests itself at the post-anthesis growth stage during which it can be assessed visually using standardized scores for retention of green-leaf area [5]. However, because the trait is expressed at the post-flowering growth stage, significant time and material resources are required to phenotype ‘stay-green’ in cereals. Although some bi-parental QTL studies have reported the co-location of QTL for delayed senescence, grain yield, and biomass yield in sorghum, knowledge of the genetic mechanisms behind this pleiotropy is limited [6]. Regardless, this pleiotropy or possibly genetic linkage between an economically important trait such as grain yield, whose assessment is relatively cumbersome, and an easily assessed trait such as delayed senescence implies that indirect selection may be used to increase the overall efficiency in breeding programs.

In cowpea, an important legume crop for subsistence farmers of semi-arid regions of sub-Saharan Africa, South America, and Asia, improvement of grain yield under drought stress is a key objective in breeding programs. Gwathmey et al. [7], [8] demonstrated a link between late-season delayed senescence (DLS), a trait similar to ‘stay-green’, and grain yield in cowpea. More recent studies have described field and greenhouse protocols to screen for delayed senescence at the seedling stage in cowpea [9], [10], [11]. Among the traits evaluated, visual scoring of leaf senescence and maintenance of stem greenness were highly reproducible under greenhouse and field conditions at the seedling and early vegetative stages and were correlated with ability to survive and recover from prolonged drought stress [10]. Subsequently, highly reproducible QTL for this trait were mapped in a cowpea recombinant inbred line (RIL) population ‘IT93K503-1 × CB46’ in which 10 QTL regions, *Dro-1*– *Dro-10*, were identified on a genetic linkage map using both greenhouse and field-based phenotyping [11].

Despite the critical role of cowpea in sustaining the food systems of many parts of the developing world, until recently very limited genomic resources have been developed. However, a high-throughput SNP genotyping platform has been implemented for cowpea, enabling the construction of a high density consensus genetic linkage map [12], [13]. This map was constructed initially from six RIL populations with a total of 928 single nucleotide polymorphism (SNP) markers, which were used to establish syntenic relationships of cowpea with soybean (*Glycine max*) and barrel clover (*Medicago truncatula*) [12]. This resource has been improved using 11 populations and including 1107 markers [13], and is being applied to both bi-parental QTL mapping and linkage disequilibrium (LD) mapping in cowpea. Advantages of LD-based association mapping studies have been described in detail elsewhere [14], [15]. The ability to complement LD mapping with bi-parental QTL mapping provides advantages in giving confirmation to significant marker-trait associations by cross-referencing with genomic regions covered by known QTL with resolutions comparable to fine-scale mapping [15].

In this study, a 1536-multiplex SNP genotyping platform was applied to cowpea germplasm originating from diverse geographical locations and breeding programs together with the consensus genetic map developed from six constituent RIL populations to (i) determine the extent of linkage disequilibrium in cowpea and (ii) detect QTL related to early and late-season delayed senescence, grain and biomass yield. Further, we compared map locations for QTL identified previously for seedling drought tolerance [11] and presently for grain yield in the RIL population IT93K-503-1 × CB46.

## Results

### Genotyping

Based on the Illumina GoldenGate assay 1,080 SNP markers had robust performance across the entire panel of 383 genotypes. Of these 1,054 SNPs had call rates ≥99% and only 14 SNPs had missing data between 5 and 10%. From the collection of diverse genotypes evaluated for these SNPs, 98.5% had call rates ≥99%. The remaining genotypes had call rates between 91.4 and 96% for the 1,080 SNPs. Genotyping results for the RIL population were reported previously [12].

### LD Analysis

For inter-chromosomal LD, 722 of the 281,731 pairwise correlations between markers exceeded the observed threshold of r^2^ = 0.25. This represented 0.26% of all possible correlations. Of these, only 30 exceeded r^2^ = 0.4 representing 0.01% of possible correlation and only two correlations were above r^2^ = 0.5 (r^2^ = 0.5001 and 0.561963). Therefore, 99.7% of r^2^ values for unlinked markers fell below the set threshold. These statistics excluded SNP marker 1_0069 that mapped at the 39.2 cM-locus on linkage group (VuLG11) of the consensus map. This marker had high r^2^ values with markers on VuLG3 (up to 0.70) but had negligible correlation with neighboring markers, which suggested a possible map error given that there was also >3 cM distance between the marker and adjacent markers on the map. Therefore, marker 1_0069 was excluded from all subsequent analyses.

With a few exceptions, intra-chromosomal r^2^ values for LGs 1, 5, 6, 8, 9, 10, and 11 decayed completely within 10 cM. LD for VuLG4 also decayed within 10 cM with the exception of 8 correlations that were above the 0.25 threshold up to distances of 50 cM. For VuLG7, LD decayed within 7 cM with the exception of 5 correlations that exceeded the threshold up to a distance of 17 cM. VuLGs 2 and 3 exhibited the highest level of LD persistence with 19 and 24 pairwise r^2^ values, respectively, exceeding the 0.25 threshold at distances greater than 10 cM. In total, 74 pairwise correlations had r^2^ values greater than the 0.25 threshold over 10 cM, representing 0.46% of all cases for all 11 LGs. The r^2^ values plotted against map distance for each linkage group are illustrated in Fig. S1.

Based on LOESS curve and r^2^ threshold intercept, LD decayed between 0 and 2 cM for VuLGs 1, 2, 3, 4, 6, 8, and 10. For VuLGs 5, 7, 9, and 11 there was no intercept between the LOESS curve and the 0.25 threshold, suggesting that LD generally decayed within sub-cM distances for these four LGs (Fig. S1).

### Population Structure

Although the number of markers between16 and 612 generally resulted in the same profiles of mean ln likelihood values plotted against K, 187 markers were chosen for the population structure analysis based on optimal allocation of genotypes to individual subpopulations. Using the model and run parameters described in methods and plotting the resulting mean ln likelihood versus K values, there was no significant change in ln likelihood values beyond K = 24 (Fig. S2), regardless of the number of markers used in the analysis. Also, the value of alpha was most stable for K greater than 24. K = 27 was chosen for further analysis as it had the highest ln (likelihood) of all K-values. The average genetic distance between individuals in the same sub-population was 0.1128 at K = 27, and the number of individuals in each sub-cluster ranged from 4 to 206.

### Phenotyping

In all, 41 separate observations were recorded from 13 experiments encompassing grain yield, grain yield components, biomass yield, seedling-stage senescence, early vegetative senescence and post-flowering senescence (Table S1). Based on analysis of variance, ANOVA, all experiments, with the exception of grain yield in Senegal 2008, showed significant variation of all traits within populations used in the respective experiment (Table S2). Notably, post-flowering senescence was positively correlated with grain yield, pod weight, 100-seed weight, and biomass yield, in Nigeria_2007, Senegal_2008, and Burkina Faso_2008. However, exceptions were observed in Burkina Faso_2008 where post-flowering senescence was negatively correlated with biomass (Table 1).

**Table 1:** Spearman Rank correlations and corresponding p-values for mean phenotypic values of diverse cowpea genotypes evaluated under drought stress in three experiments.

	PFS	Grain yield	Biomass	Seed weight
Nigeria 2007 (number of genotypes = 339)				
Grain yield	0.85****			
Biomass	0.86****	0.94****		
Pod weight	0.87****	0.98****	0.94****	
Senegal 2008 (number of genotypes = 155)				
Grain yield	0.34****			
Biomass	0.34****	0.05^ns^		
100-seed weight	−0.05^ns^	0.13^ns^	0.10^ns^	
Burkina Faso (number of genotypes = 187)				
Grain yield	0.49****			
Biomass	−0.26***	0.11^ns^		
100-seed weight	0.53****	0.79****	0.08^ns^	
Pod number	0.58****	0.82****	0.07^ns^	0.84****

ns = not significant at 0.05 level,

*** = p≤0.001,

**** = p≤0.0001. PFS = post-flowering senescence.

### Bi-parental QTL Mapping

**Figure 1:**
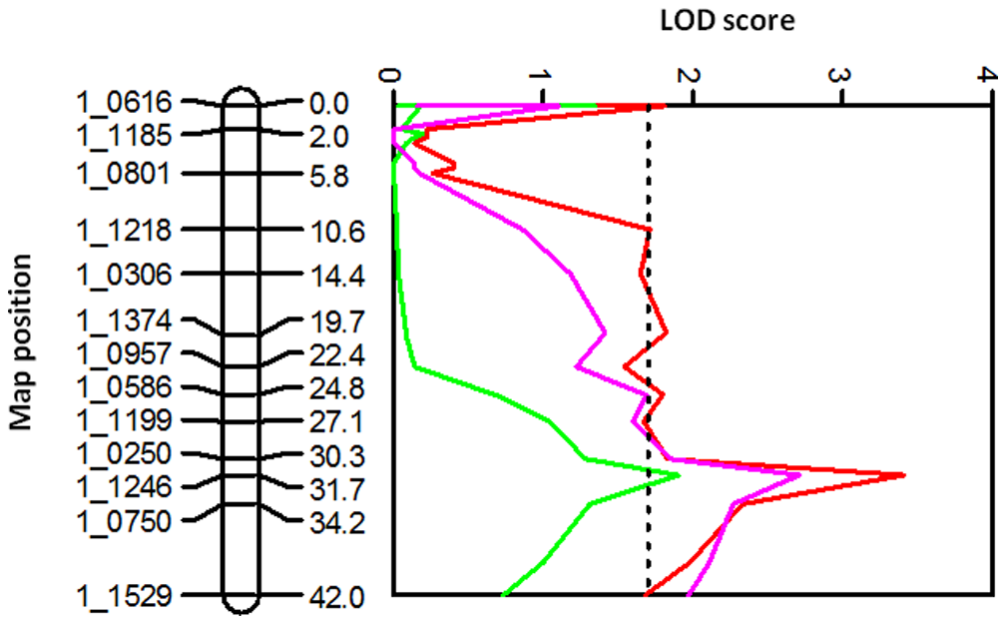
Co-location of delayed senescence (green) and grain yield (red and magenta) on QTL *Dro-3.* Vertical broken lines represent significance thresholds based on 1000 permutations at the 0.05 significance level. Delayed senescence LOD profile was based on reanalyzed data from a greenhouse experiment reported in Muchero et al. (2009a) study, grain yield data was derived from Senegal 2008 (red) and USA 2007B (magenta) experiments.

**Figure 2:**
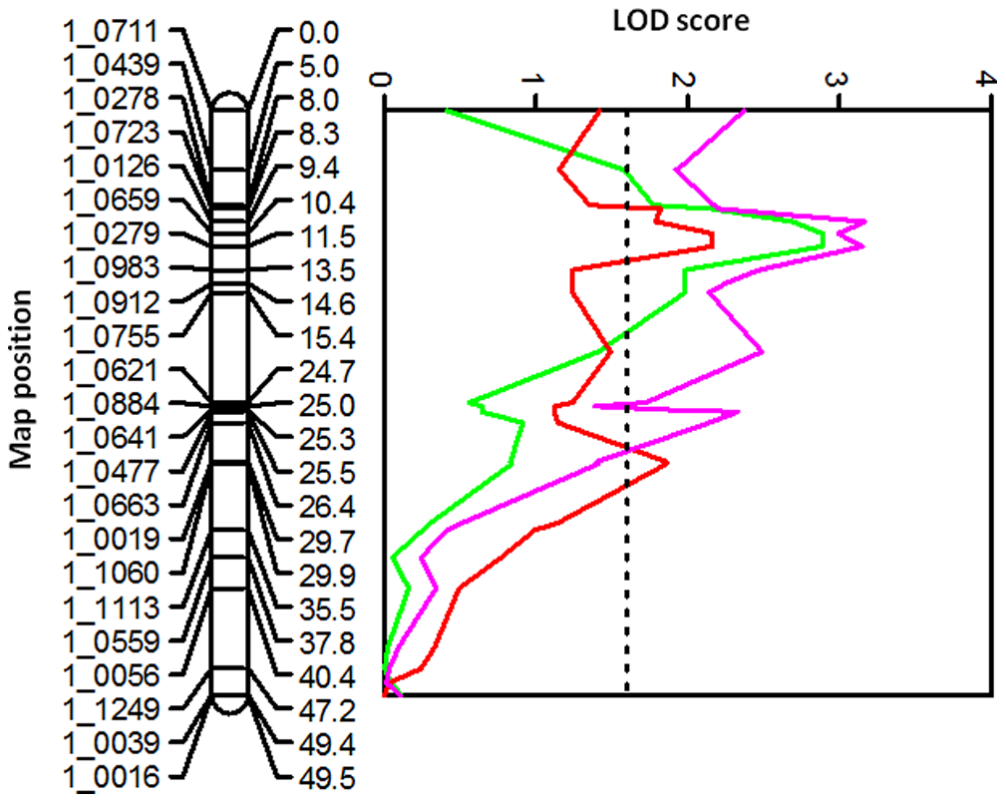
Co-location of delayed senescence under greenhouse conditions (green), grain yield from the USA 2007A experiment (red), and 100-seed weight from the Senegal 2008 experiment (Magenta) on QTL *Dro-1* in the cowpea RIL population IT93K-503-1 × CB46. Vertical broken lines represent significance thresholds based on 1000 permutations at the 0.05 significance level.

In the RIL population (IT93K-503-1 × CB46), 6 QTL for grain yield were identified across locations, reaching the more stringent 0.005 Kruskal-Wallis significance level in at least one experiment (Table 2). Although not detected in every experiment, all six QTL were identified across locations in Burkina Faso, Senegal, and the USA and coincided with QTL for delayed senescence at the seedling or early vegetative stage previously identified in the RIL population under greenhouse and field conditions [11]. Therefore, QTL designations from the previous study were used in the current study. Of the six QTL identified for grain yield, four were associated with biomass measured in Burkina Faso and Senegal. Two of these, *Dro-7* and *Dro-8,* met or exceeded the 0.005 threshold for QTL declaration (Table 2). QTL *Dro-3* detected on AFLP LGs 2 (later resolved to be two separate LGs, 8 and 11 of the IT93K-503-1 × CB46 SNP genetic map, and corresponding to consensus VuLG8 and VuLG11 respectively), had the strongest expression across experiments. QTL *Dro-3* was identified in all experiments conducted in Burkina Faso, Senegal, and USA with significance levels reaching 0.001, 0.0001, and 0.001, respectively. This was followed by QTL *Dro-7* on LG9 (corresponding to consensus VuLG1), which was identified in all locations with at least three experiments achieving the 0.01 significance level or higher. In general, LOD profiles for grain yield and delayed senescence at the seedling or early vegetative stage demonstrated similarity across entire linkage groups. Examples of LOD trace profiles for delayed senescence measured under greenhouse conditions and grain yield measured under field conditions are shown in Fig. 1 (*Dro-3*) and Fig. 2 (*Dro-1*). In each case the allele for delayed senescence was associated with increased grain yield and (or) biomass yield.

**Table 2:** Grain and biomass yield QTLs identified in the cowpea recombinant inbred line population IT93K-503-1 × CB46.

QTL name	Consensus map position:LG (cM range)	IT93K-503-1 × CB46 mapposition: LG (cM range)	Kruskal-Wallis significance level							
			Burkina Faso		Senegal		USA 2007A	USA 2007B	USA 2007C	USA 2008A
			GY^b^	BY^b^	GY	BY	GY	GY	GY	GY
*Dro-7*	1 (12.2 – 33.1)	[6] **9** a (0–20.5)	0.005	0.0005	0.05	–	0.05	0.01	0.1	0.001
*Dro-10*	3 (60.5–70.8)	[10] **1** (93.1 – 110.9)	0.05	–	0.001	–	0.05	–	–	0.05
*Dro-8*	4 (34.3–59.3)	[7] **6** (8.2–67.4)	0.0005	0.005	0.01	–	0.05	0.05	0.05	0.05
*Dro-1*	7 (8.8–34.5)	[1] **3** (0–29.9)	0.05	0.001	0.001	–	0.005	0.01	0.05	0.005
*Dro-3*	8(33.7–48.6)	[2] **8** (10.6–42.0)	0.001	0.05	0.0001	–	0.001	0.005	0.005	–
*Dro-3*	11 (2.6–43.0)	[2] **11** (46.6–48.3)	0.05	–	0.005	0.05	–	–	0.05	–

a Linkage groups (LG) in brackets correspond to the AFLP-only map (Muchero et al. 2009a); LGs in bold correspond to the SNP-only map (Muchero et a l. 2009b) and the cM range of the delayed senescence QTL remapped on the SNP-only map.

b GY = Grain yield; BY = Biomass yield.

### Association Mapping

Significance thresholds (α_G_) calculated for the MLM analysis using the Simple*M* and FDR methods gave similar results. With a M_eff_ of 203 determined by the Simple*M* method, an adjusted significance threshold was calculated as: α_G_ = 0.05/203 = 2.46×10^−4^. The highest FDR-based significance threshold was calculated as α_G_0.2 (1/856) = 2.34×10^−4^. Seven loci, comprising 16 SNPs, exceeded the significance thresholds in at least one experiment (Table 3). Since associations with p-values less than 0.01 represented only 1.5% of all associations, we reported associations falling below the Simple*M* and FDR significance thresholds but with p-values less than 0.01for each locus reported in Table 3. QTL intervals ranged from 0 to 3.2 cM with percent phenotypic variance explained ranging from 0.8 to 9.5% (Table 3). Of the seven loci, three co-located with delayed senescence QTLs *Dro-1*, *Dro-3*, and *Dro-7* representing three of the six QTL observed using bi-parental QTL mapping (Table 2). Two loci on VuLGs 4 and 7 which exhibited the highest level of significance were both associated with senescence, biomass yield, and grain yield. These two loci were detected under greenhouse screening for delayed senescence as well as field-based phenotyping for grain and biomass yield in Burkina Faso, Senegal, and USA (Table 3). The VuLG7 locus encompassed 3.2 cM and corresponded to QTL *Dro-1* that was initially reported in Muchero et al [11] where the same locus had the highest level of significance and reproducibility in that study as well. It is noteworthy that the markers associated with the QTL peak *Dro-1* in the RIL population, 1_0983 and 1_0279 (Fig. 2), also had high trait associations based on diverse genotypes (Table 3), suggesting that this locus harbors bona fide trait determinant(s) of the stay-green trait. Five of the seven loci exhibited evidence of pleiotropy and were associated with at least two of the three traits (Table 3) evaluated in this study. Table S3 shows suggestive QTLs that failed to meet significance thresholds in any experiment but had p-values of association <0.01 in at least two instances.

**Table 3:** Genomic loci associated with delayed senescence, grain yield, grain yield components and biomass yield in cowpea based on Mixed Linear Model association mapping and synteny with reference legume genomes.

Trait_Experiment	Marker	LG	Position(cM)	P-value	r^2^	QTL	*Phaseolus vulgaris* Chromosome_start postion	*Glycine max* Chromosome_start position
Seed weight per plant_BF 2008B	1_0029	1	18.420	1.62E-04	0.050	*Dro-7*	5_39290868 (5.7e-29)	15_1274309 (1.3e-8)
Pod weight per plant_BF 2009C	1_0029	1	18.420	2.90E-03	0.018			
100-seed weight_BF 2009A	1_0029	1	18.420	4.70E-03	0.023			
Biomass_BF 2009C	1_0029	1	18.420	4.90E-03	0.026			
Seed weight per plant_BF 2009C	1_0029	1	18.420	5.90E-03	0.016			
Biomass_BF 2008	1_0589	2	17.046	2.21E-04	0.049		7_47769161 (6.9e-47)	10_32269550 (8.1e-43)
Grain yield_BF 2008	1_0589	2	17.046	1.40E-03	0.044			
Senescence_Greenhouse 2	1_0067	3	10.556	1.59E-04	0.057		2_43425949 (2.4e-46)	13_41927008 (2.6e-36)
Senescence_Greenhouse 1	1_0067	3	10.556	5.00E-03	0.019			
Senescence_Greenhouse 1	1_0206	4	0.009	7.71E-05	0.039		5_516531 (1.5e-42)	4_12399615 (1.8e-38)
Biomass_Senegal 2008	1_0296	4	0.009	4.10E-03	0.038		3_43338952 (5.7e-48)	5_3321676 (5.4e-45)
Seed weight per plant_BF2009A	1_0888	4	0.455	4.04E-04	0.056		5_794965 (1.0e-6)	5_33610435 (4.19e-2)
Seed weight per plant_BF2009C	1_0888	4	0.455	8.42E-04	0.052			
Pod weight per plant_BF2009C	1_0888	4	0.455	2.10E-03	0.019			
Grain yield_USA 2007C	1_0049	5	9.456	1.92E-04	0.095		1_859379 (1.3e-43)	17_41145050 (2.6e-36)
Grain yield_USA 2007A	1_0049	5	9.456	5.70E-03	0.059			
Senescence_BF 2008	1_0108	7	15.844	6.76E-04	0.023	*Dro-1*	2_7436099 (2.1e-15)	9_39304124 (2.3e-5)
Biomass_BF 2008	1_1150	7	15.844	3.60E-03	0.033		2_7409687 (2.1e-34)	2_3284032 (4.5e-8)
Grain yield_BF 2008	1_1150	7	15.844	7.90E-03	0.031			
Senescence_Greenhouse 1	1_0279	7	18.213	2.70E-03	0.019		2_27942808 (1.2e-37)	9_39955414 (1.7e-32)
Seed weight per plant_BF 2009B	1_0279	7	18.213	7.30E-03	0.025			
Senescence_USA 2009	1_0983	7	19.081	9.12E-05	0.070		2_27089201 (6.5e-41)	9_40434989 (1.2e-21)
Senescence_Greenhouse 1	1_0983	7	19.081	2.40E-03	0.022			
Grain yield_Nigeria 2007	1_0140	10	34.896	4.52E-04	0.015	*Dro-3*	10_30910809 (3.1e-51)	1_46091028 (2.2e-37)
Senescence_Nigeria 2007	1_0140	10	34.896	9.00E-03	0.008			
Seed number per pod_BF 2009C	1_0759	10	35.578	8.20E-03	0.033		10_29466207 (1.3e-43)	1_45401915 (7.6e-18)
Grain yield_Nigeria 2007	1_0759	10	35.578	9.40E-03	0.009			
Senescence_USA 2009	1_1405	10	37.373	2.14E-04	0.063		10_16853286 (3.4e-38)	1_44037621 (2.3e-24)
Pod number per plant_BF 2009A	1_1405	10	37.373	5.70E-03	0.033			

### Pleiotropy between Delayed Senescence, Grain Yield, and Biomass Yield

**Figure 3:**
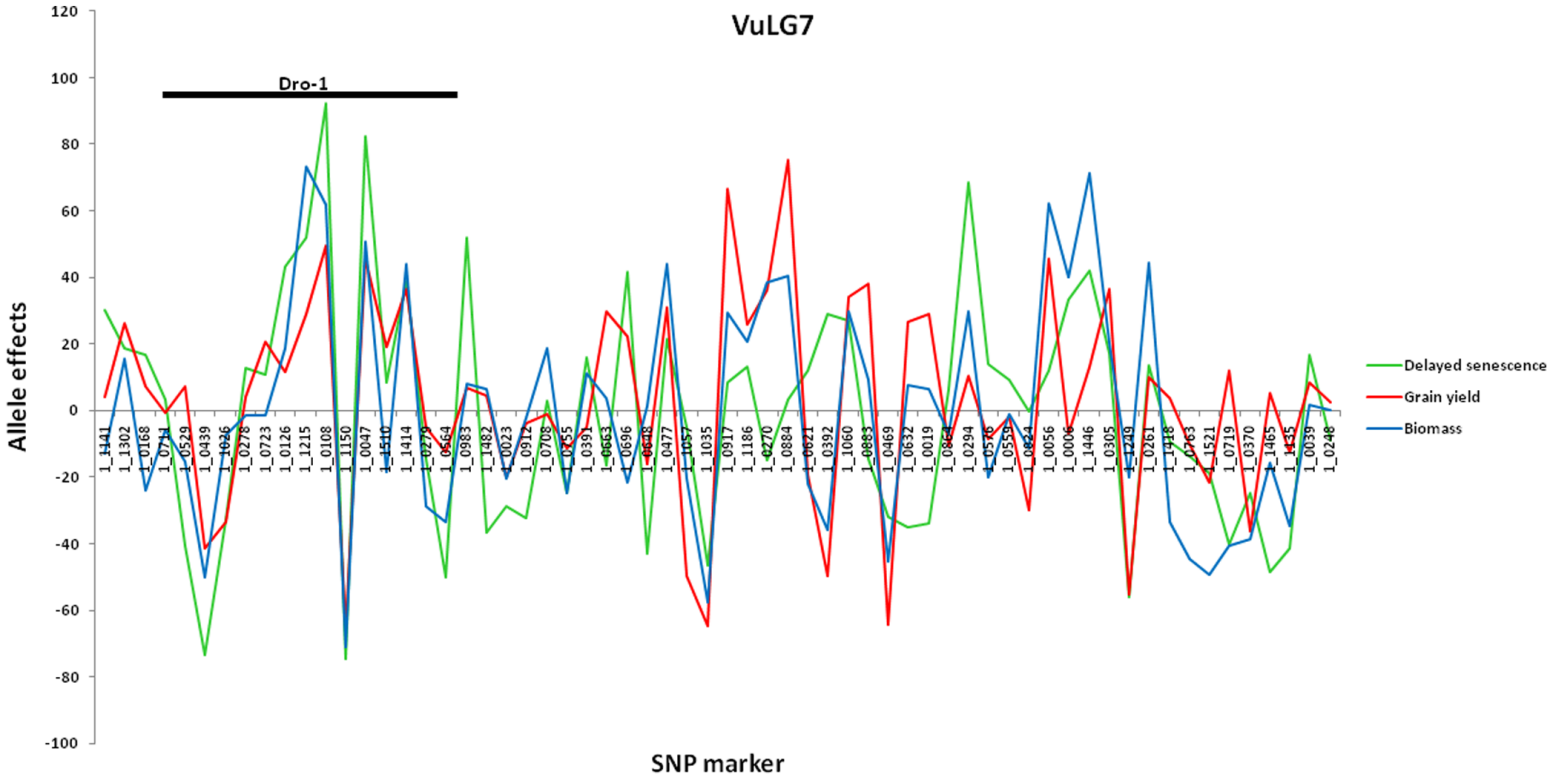
MLM analysis-derived allele effects for SNP markers across VuLG7 for delayed senescence, grain yield, and biomass phenotypes based on 187 diverse genotypes evaluated in Burkina Faso (BF 2008). Allele effects were scaled on a percent basis relative to the highest value for each phenotypic parameter.

At the phenotypic level, pleiotropy between the three traits (stay-green phenomenon) was suggested by significant correlations among mean phenotypic values for delayed senescence, grain yield, grain yield components, and biomass yield in experiments conducted in Burkina Faso, Nigeria, and Senegal (Table 1). However, as stated above, the positive correlations were not consistent across all environments for biomass and delayed senescence phenotypes. QTLs *Dro-1*, *Dro-3*, and *Dro-7* were identified using both association mapping and bi-parental QTL mapping (Table 2 and 3) and in each case the QTLs were associated with more than one trait. It is noteworthy that the *Dro-1* QTL was also detected in Burkina Faso_2008 where delayed senescence and biomass were negatively correlated at the phenotypic level. However, allelic effects at this locus were positively correlated as would be expected with the stay-green trait (Fig. 3). Additional evidence of pleiotropy was based on co-location of QTLs at SNP markers with significant effects as described above. This co-location was observed in the RIL population as well as in the panel of diverse germplasm. In this case, profiles of LOD scores from bi-parental QTL mapping for delayed senescence, grain and biomass yield were nearly identical across linkage groups (Figs 1 and 2). This observation was further validated based on Spearman rank correlation analysis of p-values from MLM analysis, in which correlation coefficients were 0.29< r <0.63 (p = 0.000) among delayed senescence, grain yield, and biomass yield compared to 0.0347≥ r ≥0.0005 (p≥0.3299) for flowering time against delayed senescence, biomass, or grain yield.

Significant positive correlations of allele effects obtained from the MLM analysis for each of the 794 mapped and 62 unmapped SNPs suggested that the predominant pleiotropic effect was positive in which delayed senescence conferred higher biomass and grain yield, although a single instance of significant negative correlation was observed between delayed senescence and biomass in Burkina Faso (Table 1). Fig. 3 shows scaled allele effects for SNP markers on VuLG7, in part corresponding to locus *Dro-1* identified in both bi-parental and association mapping. In this case allele effects were determined based on the MLM analysis using 187 diverse genotypes in the Burkina Faso (BF_2008) experiment. The same trend was observed for data collected in Nigeria and Senegal where the three traits were evaluated in the same experiment. Allele effects for the 794 mapped SNPs were positively correlated in each of the three environments: Nigeria (0.8616≥ r ≥0.6320, p = 0.0000), Burkina Faso (0.7067≥ r ≥0.2473, p = 0.0000), and Senegal (0.1880≥ r ≥0.0748 (p≤0.0352).

### SNP Positions in *Phaseolus vulgaris* (Common Bean) and *Glycine max* Genomes

Fourteen ESTs harboring SNPs with phenotypic associations had significant BLAST matches in common bean and (or) soybean genome assemblies (Table 3). Based on that analysis, QTLs *Dro-1* and *Dro-3* had strongest support based on multiple cowpea ESTs mapping in the same contiguous regions in both common bean and soybean. QTL *Dro-3* exhibited the most collinearity with all 3 ESTs mapping in the same order on common bean chromosome 10 and soybean chromosome 1 (Table 3) encompassing 14.1 Mb and 2.1 Mb of sequence, respectively. Markers within the *Dro-1* QTL mapped on common bean chromosome 2 and encompassed a 20-MB region. In soybean, 3 of the 4 markers associated with *Dro-1* QTL mapped to chromosome 9 encompassing a substantially smaller region of 1.1 Mb.

## Discussion

The general decay of LD within distances less than or equal to 2 cM in cowpea suggested that genome-wide association mapping is practical in cowpea with the potential to provide QTL resolution up to 10× greater than bi-parental QTL mapping. Given the total length of the cowpea genetic map distance (680 cM) and the conservative 1 cM distance for complete LD decay, the cowpea consensus map should provide sufficient coverage for QTL detection. Therefore, the cowpea consensus map and its recently improved version [12], [13] with average marker distance of 0.61 cM can serve as a platform for high resolution association mapping. Furthermore, with LD generally decaying within 2 cM and only 1.30% of pairwise correlations exceeding the 0.25 threshold at distance greater than 2 cM, high resolution QTL mapping should be possible in cowpea. Instances of LD persisting in the order of cM distances is characteristic of self-pollinating species and observations reported here agree closely with observations made in other plant systems with similar reproductive behavior [16].

Using the genome-wide scan with 856 SNP markers, we were able to identify seven loci with significant marker-trait associations. Of these, five were associated with more than one trait and three of them mapped in QTL intervals that were associated with delayed senescence at the seedling stage as well grain and biomass yield in the RIL population. The co-location of QTLs for at least two of the three traits is suggestive of common genetic determinants as well as the existence of the pleiotropic stay-green phenomenon in cowpea. This was further supported by positively correlated allele effects, and largely correlated mean phenotypic values for delayed senescence, grain yield, grain yield components and biomass yield in experiments conducted in Burkina Faso, Nigeria, and Senegal. However, there was also evidence suggesting genotype × environment interaction for this trait with the Senegal location exhibiting the lowest levels of correlation between delayed senescence, biomass, and grain yield. These findings suggest the importance of the stay-green trait in cowpea as an adaptive trait in drought-prone environments. The stay-green trait must have been favored in the arid and semi-arid environments of sub-Saharan Africa where cowpea was domesticated and has been cultivated for thousands of years. The coupling of mechanisms conferring drought tolerance, grain yield, and biomass yield would confer fitness while minimizing energy usage by utilizing the same genetic pathways for survival and productivity.

Further, the fact that these QTLs could be identified across different agro-ecological environments in both West Africa and North America indicates that these loci represent viable targets for the genetic improvement of cowpea production in arid and semi-arid regions. The apparent positive pleiotropy observed here suggests that genomic selection-based approaches to introgress more than one locus at a time [4] offer the best opportunity for maximizing cowpea drought tolerance, grain yield and biomass accumulation. In particular, QTLs *Dro-1*, *Dro-3*, and *Dro-7* are potential targets for selection given their robust detection in more than one agro-ecological zone within both the RIL population and the panel of diverse germplasm. In addition to biomass and grain yield phenotypes, all three QTLs were previously reported to mediate delayed senescence at the early vegetative stage in both field and greenhouse experiments [11]. In that study, the *Dro-1* QTL had the largest percent phenotypic variance explained among 10 putative QTLs. The same interval was associated with early vegetative delayed senescence under greenhouse and field conditions in the USA as well as post-flowering delayed senescence under field conditions in Burkina Faso. Although overall biomass yield was negatively correlated with the post-flowering delayed senescence in Burkina Faso_2008, allele effects for SNP markers within the *Dro-1* interval were positively correlated for delayed senescence, biomass, and grain yield. This observation suggests that *Dro-1* QTL still exhibited positive pleiotropy and is consistent with the putative stay-green effect, regardless of trends in the overall phenotypes. Building on the present findings, genotypes with the most comprehensive constitution of favorable QTLs can be identified, including those with complementary sets of QTLs, and can be used in ideotype-driven selection schemes for improvement of grain yield, biomass yield, and drought tolerance in cowpea. High-throughput SNP genotyping in marker-assisted recurrent selection or whole genome selection breeding approaches can facilitate the required multiple QTL selection [3]. In addition to marker-assisted breeding, results of this study suggest that delayed senescence during the early vegetative stage may be used as a rapid screen for post-flowering drought tolerance mediated by the stay-green trait. Additionally, the successful delimitation of common bean and soybean genomic regions that exhibit conserved synteny with the cowpea *Dro-1* and *Dro-3* QTLs should facilitate translational genomics among these legumes for targeted candidate gene studies.

While recognizing that the ‘stay-green’ trait does not encompass all potential drought tolerance mechanisms contributing to drought adaptation in cowpea, this study demonstrated a robust association of expression of the stay-green trait with grain yield and biomass yield under drought stress. These findings indicate that assessments of delayed senescence could be useful for indirect selection of grain yield and biomass yield under drought, especially for cost-effective preliminary assessments of very large samples of germplasm or early generation screening of breeding lines in breeding programs targeting improved drought tolerance. Such a cost-saving approach would be important for breeding programs where the time and resource requirements for grain yield phenotyping in drought environments are currently high relative to seedling or early vegetative stage high-throughput phenotyping assays. These findings in cowpea are distinct from crops such as sorghum, maize, and rice where phenotyping for this trait has only been done at the post-flowering stage under field conditions [6].

From practical and basic genetics considerations, cowpea offers an important opportunity for the dissection of the stay-green trait in legume crops and perhaps more broadly as a model for indeterminate, non-cereal crops. With its relatively simple, small, diploid genome and short generation time, rapid advances can be made in understanding this trait including synteny and translational genomics-based analysis relevant to other important crops. Notably, the close evolutionary relationship of cowpea to soybean and common bean and the demonstrated high level of synteny between their genomes [12] provide a useful avenue for translational genomics.

Our results also showed that a RIL population as small as 48 lines together with a diverse germplasm set of 96 genotypes provided enough resolution to detect the co-location of delayed senescence, grain yield and (or) biomass yield QTLs. This finding corroborates previous results where we demonstrated that subsets of 57 and 70 RILs provided nearly the same mapping resolution for the delayed senescence trait as populations of 124 and 127 RILs [11]. In contrast, studies in other systems targeting complex traits required major phenotyping efforts on large populations [17], [18] that are difficult for crops such as cowpea where resources for genetic improvement are currently limited. The extent of LD in self-pollinating crops may partly explain this, suggesting that LD and bi-parental QTL mapping approaches offer sufficient power to detect loci contributing toward complex trait expression. Having the benefit of bi-parental QTL mapping helps mitigate against inflation of false negatives based on conservative significance thresholds and the multiple correction steps typical for approaches such as a MLM analysis.

In conclusion, isolating and characterizing the genetic determinants of the stay-green trait hold tremendous opportunity for the improvement of crop productivity in light of contemporary challenges that include global warming, rapidly increasing population, the need to reduce inorganic nitrogen applications in farming systems, and increasingly constrained agricultural water resources, as well as the need to develop sustainable renewable energy sources [19]. Cowpea is well positioned biologically and practically for such initiatives.

## Materials and Methods

### Plant Material

A selection of 383 inbred cowpea genotypes (Table S4) representing accessions from different geographical regions and breeding programs constituted the core diversity set for this study. Of these, 339 were phenotyped in Nigeria, 201 in Burkina Faso, 163 in Senegal, and 205 in the USA. 166 genotypes were common across experiments in Burkina Faso, Nigeria and the USA, whereas 131 were in common across experiments including Senegal. In addition, a F_2∶9_ RIL population with 113 lines developed from the cross IT93K-503-1 (drought tolerant) × CB46 (drought susceptible) and the parental genotypes were used.

### Genotyping

Each of the 383 genotypes was SNP-genotyped using the Illumina GoldenGate 1536-SNP assay and data for allele calls at each SNP processed as described by Muchero et al. [12]. The RIL population was genotyped previously using the same assay [12].

### LD Analysis

LD decay along linkage groups was analyzed using the Graphical Genotyping (GGT2.0) software [20] based on 561 markers that represented unique loci with minor allele frequencies (MAF) >0.1 and were distributed evenly over the 11 linkage groups of the cowpea consensus genetic map of Muchero et al. [12]. The r^2^ values for all pairwise correlations between markers were plotted against map distance for each linkage group using Excel. The critical r^2^ value was determined by taking the square root of r^2^ values for all unlinked markers and taking the parametric 95^th^ percentile of the distribution [21]. Markers with unknown map positions were excluded from any LD analysis. Extent of LD decay was determined by fitting a LOESS smoothing curve to r^2^ scatter plots of each linkage group with the smoothing parameter set between 0.1 and 0.2 using Statistix 9.0 software [22]. The cM distance at which the curve intercepted the r^2^ threshold was considered to be the extent of LD on the respective linkage group.

### Population Structure

Population structure was evaluated using the software STRUCTURE 2.1 [23]. Appropriate marker number for the determination of population sub-structuring was determined by empirical evaluation of 16, 67, 102, 187, 317, and 612 markers selected based on map distances and coverage of the consensus genetic map using an in-house Fox-Pro sampling script. Hypotheses for 1 to 40 sub-populations were tested using STRUCTURE 2.1 with admixture and independent allele frequencies using the run parameters: length of burn-in = 10,000 and replications after burn-in = 1000. The lowest K at which no significance change was observable for the mean ln(likelihood) estimates was taken as the number of sub-populations. In addition to sub-population analysis, a kinship matrix was calculated using software TASSEL 2.1 (http://www.maizegenetics.net) based on 856 markers used in the association study.

### Seedling-stage Delayed Senescence in Diverse Germplasm

**Figure 4:**
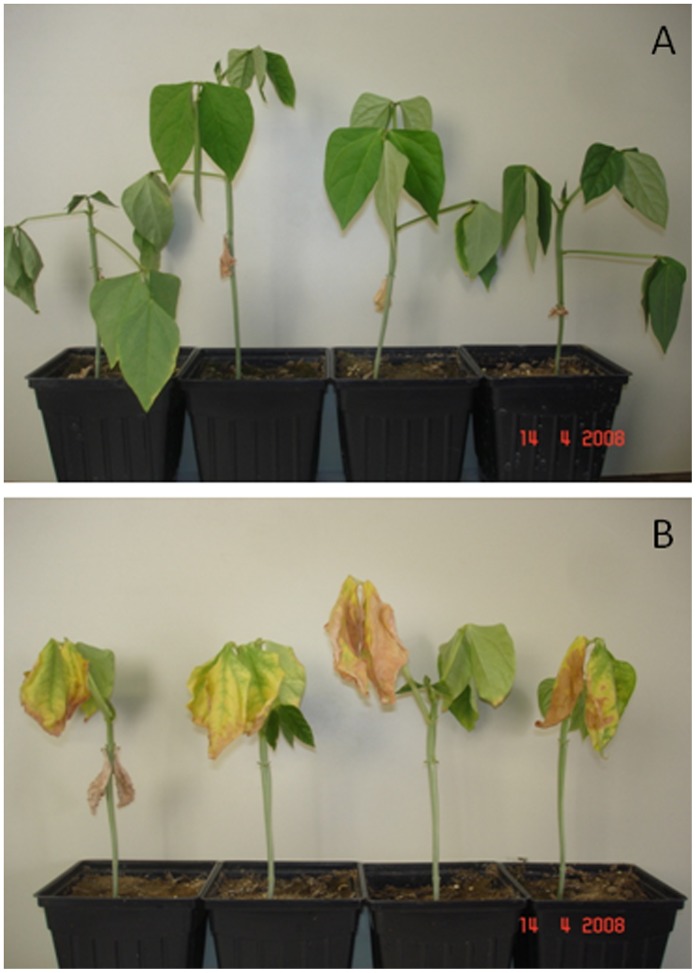
Representative phenotypes of the delayed senescence trait under greenhouse conditions illustrating (A) four replicates of a delayed senescence genotype and (B) four replicates of a senescent genotype.

The delayed drought-induced senescence trait (early vegetative delayed senescence) was evaluated on 205 genotypes in two greenhouse (Senescence_Greenhouse 1 and Senescence_Greenhouse 2) and one field (Senescence_USA 2009) experiments in the USA. Four replicates with one plant of each genotype were evaluated in greenhouse experiments. Four replicates of 1-meter plots with ten plants each were used to evaluate genotypes under field conditions. In greenhouse experiments, ratings were taken for visual-based senescence and maintenance of stem greenness on 2–6 week-old cowpea plants as described by Muchero et al. [10], [11]. Briefly, plants were rated on scale of 1–5 for leaf senescence and maintenance of stem greenness during the course of greenhouse experiments. Similarly, field-based delayed senescence was evaluated before flowering on the same set of genotypes on 4–6 week-old plants using the same protocols as described by Muchero et al. [10], [11]. Evaluations were conducted at the University of California-Riverside Coachella Valley Agricultural Research Station (CVARS) in Thermal, California, USA. Representative senescence phenotypes under greenhouse conditions are shown in Fig. 4.

### Grain Yield, Biomass Yield, and Post-flowering Senescence Phenotyping

Experiments in all locations were conducted under rainless or limited rain conditions to induce drought stress. Climatic conditions related to maximum and minimum temperatures, evapo-transpiration, and total rainfall during the course of each experiment are summarized in Table S5. A randomized complete block design was used for each experiment. In each location, number of days to 50% flowering, delayed senescence based on a 1–5 scale, biomass, and grain yield averaged over harvested plants were scored and measured. Late season delayed senescence ratings were not taken for grain yield and biomass experiments in California. Table S1 summarizes experimental details and traits evaluated in each experiment.

Briefly, 96 cowpea genotypes and 57 RILs from the IT93K503-1 × CB46 population were evaluated for grain yield at the University of California-Riverside CVARS station under three treatments: fully irrigated (USA 2007A); irrigation withheld for 15 days from the day of planting (USA 2007B); and irrigation withheld for 30 days from the day of planting (USA 2007C). Normal irrigation cycle was resumed after stress treatment. In 2008, 201 diverse genotypes and 84 RILs were evaluated for grain yield at CVARS (USA 2008).

In 2007, 339 cowpea genotypes were planted at the International Institute of Tropical Agriculture (IITA) Kano station in Nigeria (Nigeria 2007), and evaluated for biomass, grain yield, 100-seed weight, and post-flowering delayed senescence. Three replicates of 3 m×75 cm plots were planted with 15 seeds of each genotype. Pre-planting irrigation was applied to soil saturation and two additional irrigation cycles were applied at 2 and 4 weeks after planting after which no additional irrigation was applied. A total of 4 mm rainfall was recorded over the course of the experiment (Table S5). Ten plants from each plot were evaluated for post-flowering senescence and were harvested for biomass and grain yield measurements. Harvested plant materials were oven-dried to constant weight for 48 hours at 80°C before weighing.

In Burkina Faso, 201 genotypes and 49 RILs were planted at the Institute of Environmental and Agricultural Research (INERA) Kamboinse experiment station (BF 2008) and were evaluated for biomass, grain yield, and delayed senescence. The same set of material excluding the RIL population was evaluated in Burkina Faso in 3 additional experiments, BF 2009A and BF 2009B at the Pobe-Mengao experiment station, and BF_2009C at the Kamboinse experiment station. In each experiment, 2–2 m-plots replicated three times were planted with a total of 40 seeds for each genotype. Experiment BF_2008 in Kamboinse was conducted entirely under natural rainfall conditions with 123 mm and 28 mm being received for last two months of the experiment, September and October, respectively. Similarly, experiment BF_2009C was conducted under natural rainfall conditions. Planting followed a 260 mm rainfall event in late August with 98 mm being received in the last six weeks of the experiment For experiments BF_2009A and BF_2009B at Pobe-Mengao, planting was carried out after a 58-mm rainfall at the end of July for BF_2009A, and a 43-mm rainfall event for BF_2009B. A total of 324 mm of rainfall was received within 4 weeks from the beginning of August until the end of September, and 20 mm was received during the whole month of October. No additional rainfall or supplemental irrigation was received or applied for the remainder of the experiment. Delayed senescence was scored as described above and ten plants per replicate were harvested for biomass, grain yield, and grain yield components evaluation (Table S1).

163 genotypes and 51 RILs were evaluated at the Senegalese Institute of Agricultural Research (ISRA-CNRA) Bambey station in Senegal (Senegal 2008). Three replicates, each with 2–4 m long rows were planted with a total of 68 seeds. Plots were planted after a 22.7-mm rainfall event that was followed by a total of 102.3 mm rainfall during the course of the experiment (Table S5). No supplemental irrigation was applied. Delayed senescence was scored at the post-flowering stage as described above. Thirty plants per replicate were harvested and solar-dried for 15 days before being evaluated for biomass, overall grain yield, and 100-seed weight.

### QTL Mapping for Grain Yield in RIL Population

The Kruskal-Wallis and Multiple-Model QTL Mapping (MQM) packages of MapQTL 4.0 [24] were used to map grain yield QTL for the RIL population. LOD significance thresholds were determined for each linkage group using the permutation test with 1000 replicates. A QTL region was considered significant when it was detected in at least two experiments with one of the experiments meeting a Kruskal-Wallis significance of 0.005 or higher as recommended by authors of the software. Although the MQM was not used to define QTL, results from the analysis were used to verify Kruskal-Wallis results as well as for visual display of QTL positions on linkage groups.

### QTL Mapping for Delayed Senescence in RIL Population

QTLs detected for the early vegetative delayed senescence trait in the RIL population had been described by Muchero et al. [11] using an AFLP marker-based genetic linkage map. For the current study, phenotypic data sets of 113 RILs from that previous study were reanalyzed using the SNP and combined SNP-AFLP maps for the RIL population described in Muchero et al. [12]. The data sets represented six greenhouse and three field experiments. AFLP markers flanking previously described QTL intervals were used to identify QTLs *Dro-1* to *Dro-10* on current linkage groups and SNPs flanking these intervals were used to determine positions on the consensus map. LOD score profiles across linkage groups were drawn using MapChart 2.2 [25] to illustrate QTL co-location.

### Association Mapping

TASSEL 2.1 software (http://www.maizegenetics.net) was used to identify marker-trait association using a total of 856 SNPs with MAF >0.1. Of the 856, 62 SNPs were not part the consensus map and therefore their genomic position was not known. The mixed-linear model analysis (MLM) [26] was used to establish trait-marker association for delayed senescence, grain yield and biomass yield. The model utilized kinship, population structure and days to 50% flowering as covariates and this has been demonstrated to be effective in reducing Type I error rate that leads to false positive associations. However, the same analysis has been reported to increase Type II error rate resulting in biologically relevant loci failing to meet conservative significance thresholds set for dealing with multiple testing of large numbers of SNP markers [16], [27]. Loci consistently identified over experiments by the MLM analysis but falling below the significance thresholds were verified based on evidence of co-location with QTL from the RIL population and were reported as suggestive QTL.

Allele effects were determined concurrently with marker-trait associations using the MLM analysis in the TASSEL 2.1 software.

To avoid spurious association due to LD persistence, LD was determined for all loci with significant association. Two or more QTLs were considered the same when (i) the markers next to the adjacent QTLs had r^2^ greater than the threshold or (ii) unmapped markers had high pair-wise correlations with mapped loci harboring a detected QTL. For non-adjacent loci as well as loci on different linkage groups, the locus showing the highest significance was accepted as the QTL peak.

### Pleiotropy between Delayed Senescence, Grain Yield, and Biomass Yield

Pleiotropic effects between delayed senescence, grain yield, and biomass yield were accepted only when (1) QTLs coincided on the same map position, (2) statistically significant Spearman rank correlation of p-values, and (3) statistically significant Spearman rank correlation of allele effect of each marker. For unmapped markers, co-location was accepted when the same marker gave significant associations with at least two of the three traits. Further, remapping the QTLs for delayed senescence at the seedling stage was used to assess the incidence of markers within these QTL regions that also showed statistically significant association with grain yield and biomass in the association mapping study. Co-location was visualized by plotting –log (p-value) against map distance along individual linkage groups of the cowpea consensus map for each of the three traits. To rule out spurious correlations, correlation analysis was carried out using MLM-derived p-values of association for the three traits and flowering from the Nigeria 2007 dataset.

### Statistical Analysis

The Simple*M* method [28] and the false discovery rate (FDR) approach [29] were used to test for statistical significance of marker-trait associations from the MLM analysis. The Simple*M* method accounts for the lack of independence in multiple tests resulting from extensive LD between SNP markers tested for association. The program calculates an effective number of independent tests (M_eff_) which is then used as the number of independent tests in the Bonferroni correction for multiple testing [30]. In this analysis, 856 markers were used to calculate M_eff_ with PCA threshold of 0.95 and the Simple*M* source code in R statistical program (R Development Core Team (2010). The formula α_G_ = 0.05/M_eff_ was used to calculate the p-value significance for association.

For the FDR method, the p-values from the MLM analysis were subjected to the FDR method based on a false discovery rate, q* = 0.20 as described by Benjamini and Yekutieli [31].

Analysis of Variance (ANOVA) and Spearman rank correlations were calculated using Statistix 9 software [22].

### Estimation of QTL Positions in *P. vulgaris* and *G. max* Genomes

Cowpea ESTs harboring SNPs with significant associations to phenotypes were used in BLAST analysis to infer positions on the common bean and soybean reference genomes (http://www.phytozome.net). Cowpea ESTs (121 nucleotides long) with 60 bases flanking the SNP position were used in the analysis. Start positions of the most significant BLAST matches were reported.

## Supporting Information

Figure S1 **LD decay curves for eleven linkage groups of the cowpea consensus genetic linkage map.**
(PDF)

Figure S2 **Plot of mean values of ln likelihood over k-estimate of the number of subpopulations (K) inference for 383 diverse cowpea genotypes.**
(TIF)

Table S1 **Traits analyzed in Burkina Faso (BF), Senegal, Nigeria, and the United States of America (USA) and results of ANOVA analysis.**
(XLSX)

Table S2 **Suggestive marker-trait associations based on MLM analysis.**
(XLSX)

Table S3 **List of cowpea genotypes evaluated for delayed senescence, grain yield, and biomass accumulation.**
(XLSX)

Table S4 **Details of experiments conducted to evaluated cowpea response to drought stress.**
(DOCX)

Table S5 **Summary of climatic conditions for field experiments conducted Burkina Faso (BF), Nigeria, Senegal, and the United States of America (USA).**
(DOCX)
