# Supplementary figures and images for: Genetic Architecture of Delayed Senescence, Biomass, and Grain Yield under Drought Stress in Cowpea
Source: PLoS One. 2013 Jul 30;8(7):e70041. doi: 10.1371/journal.pone.0070041 (PMC3728364; doi:10.1371/journal.pone.0070041)

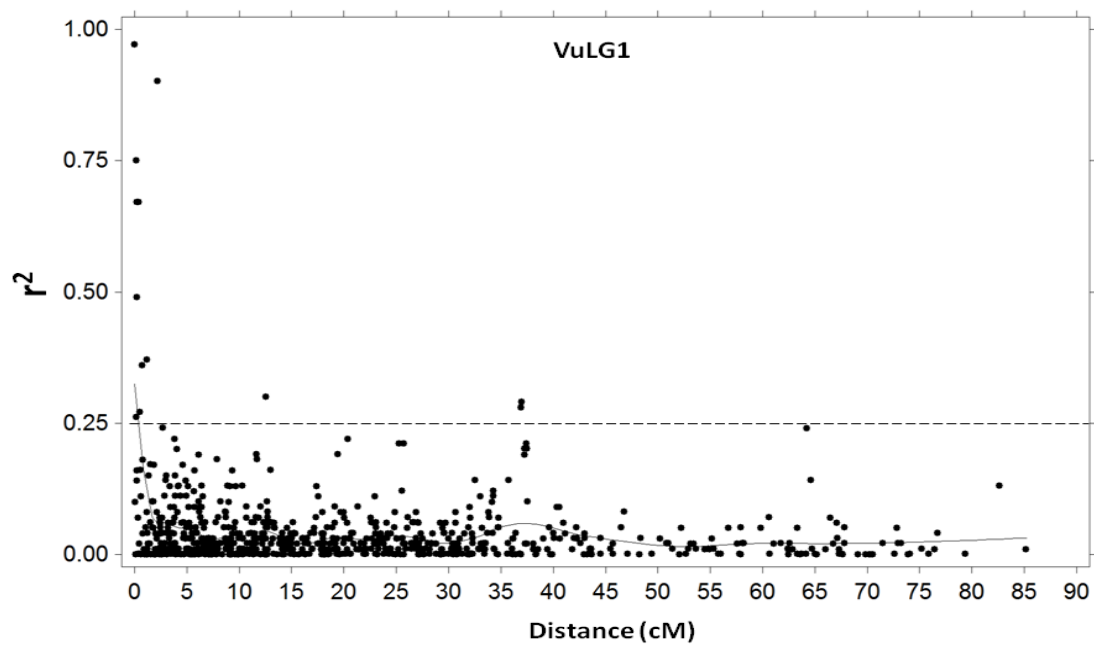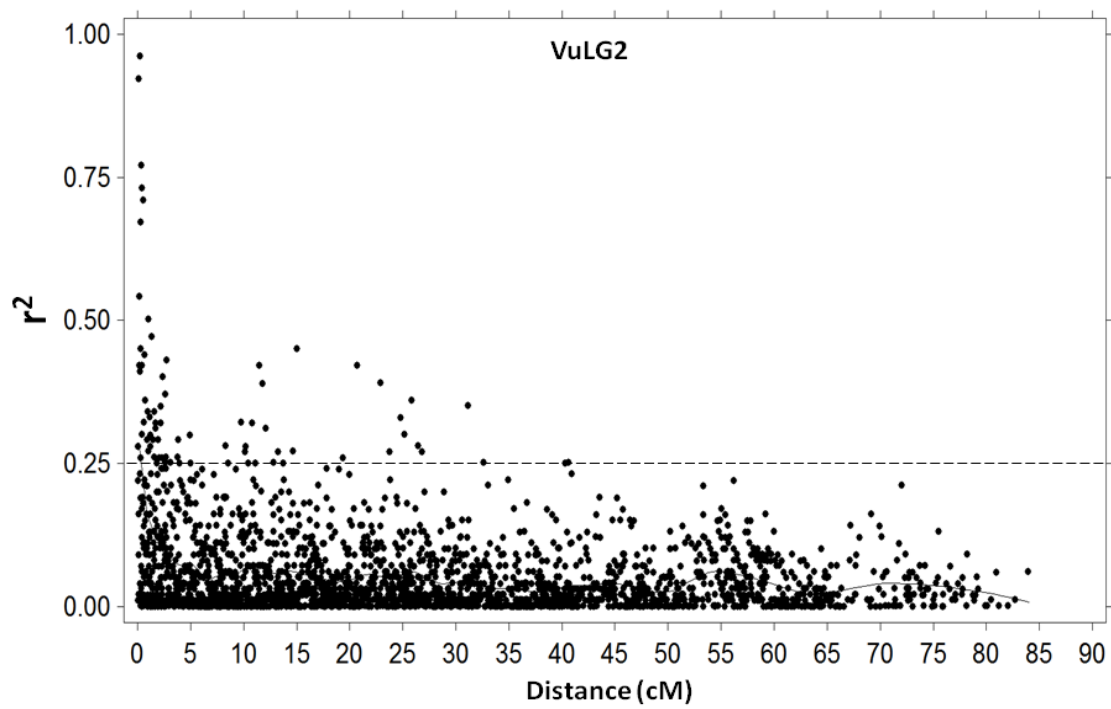

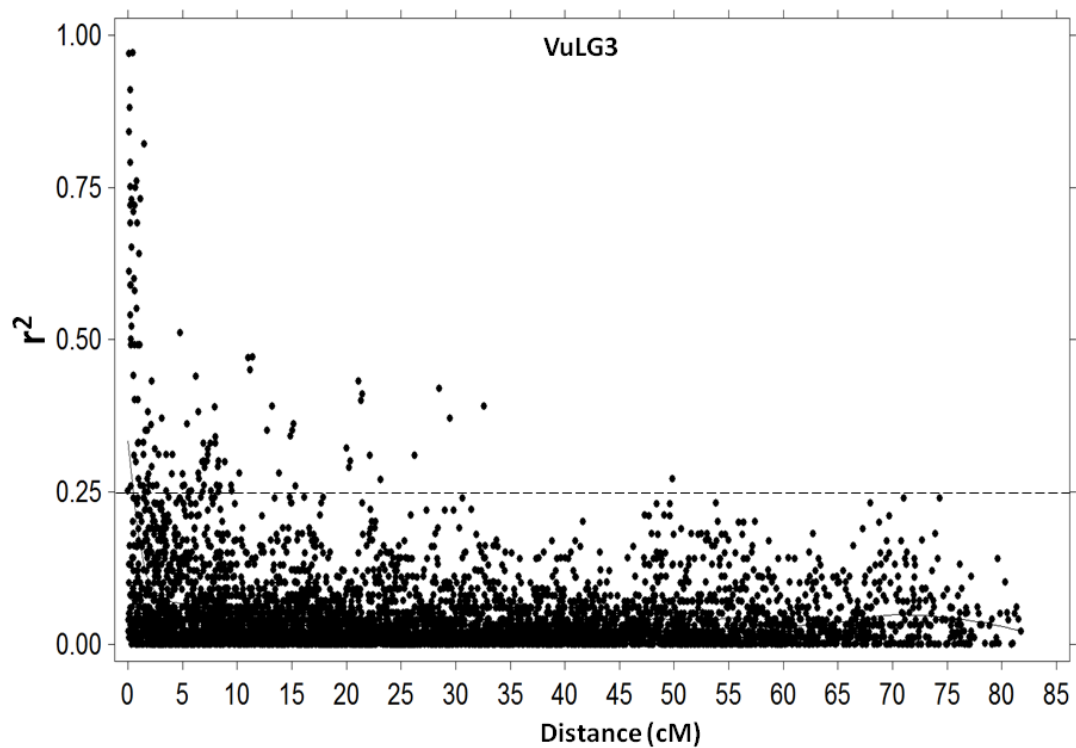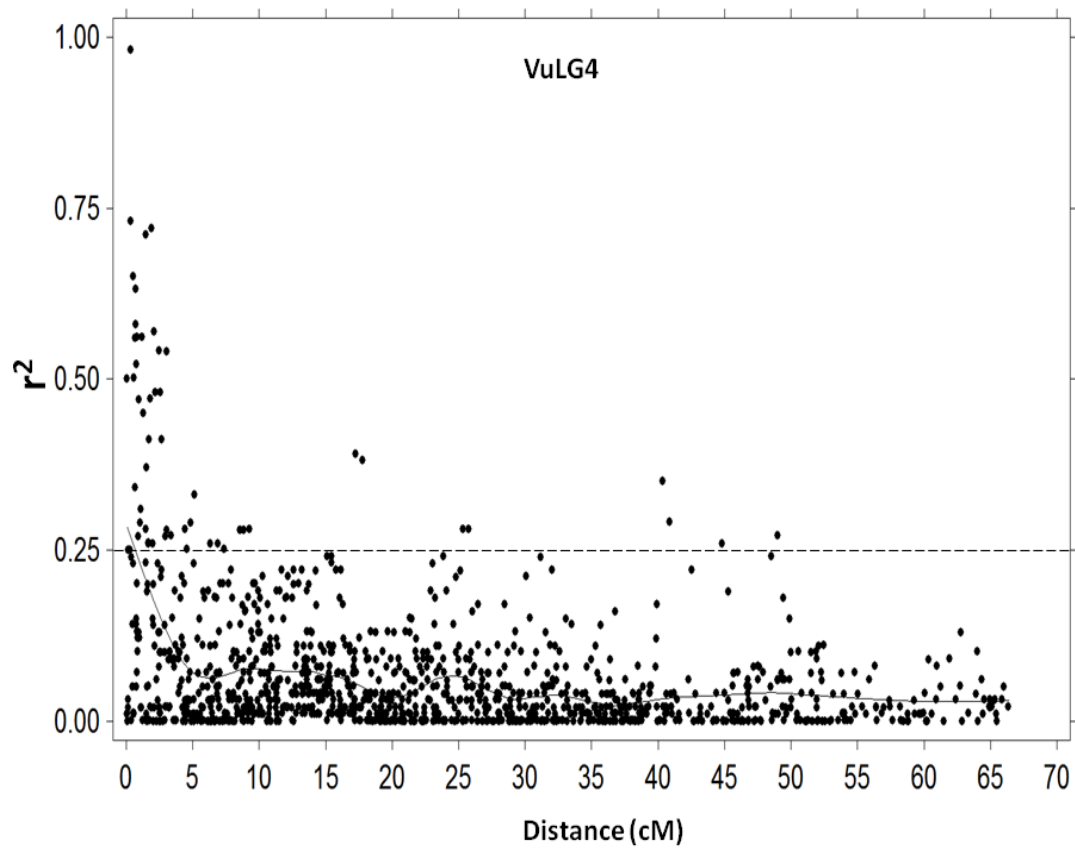

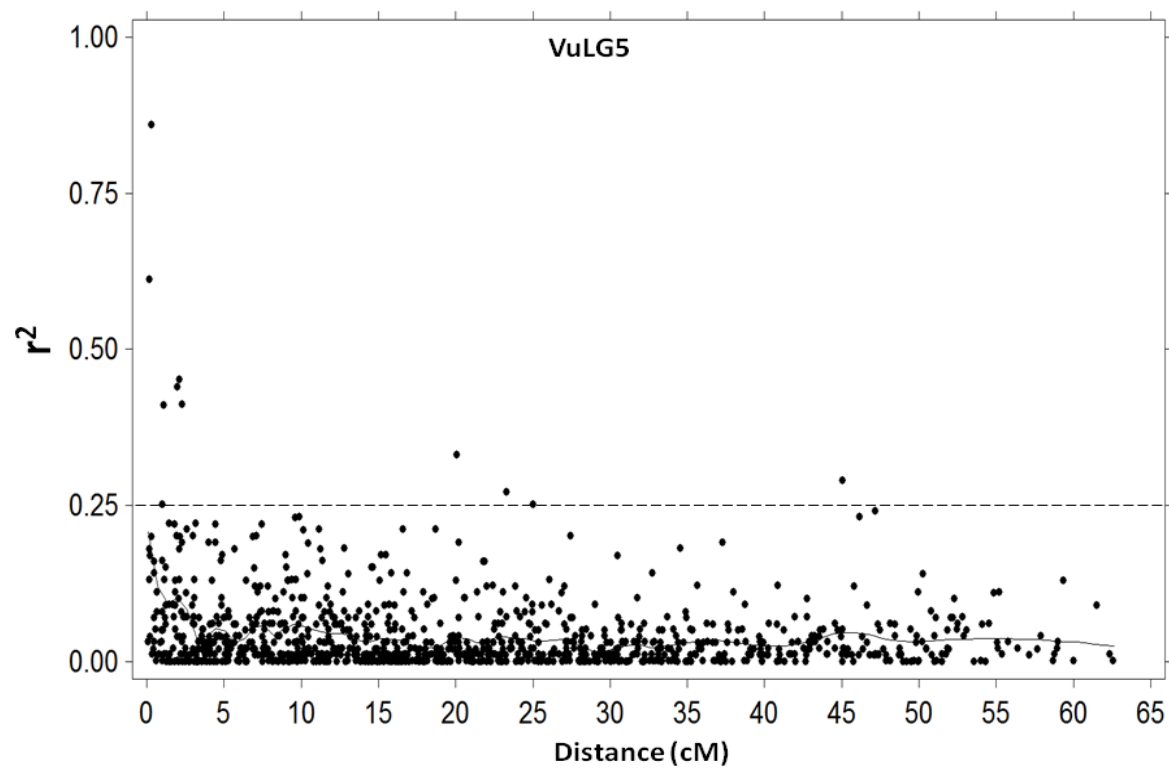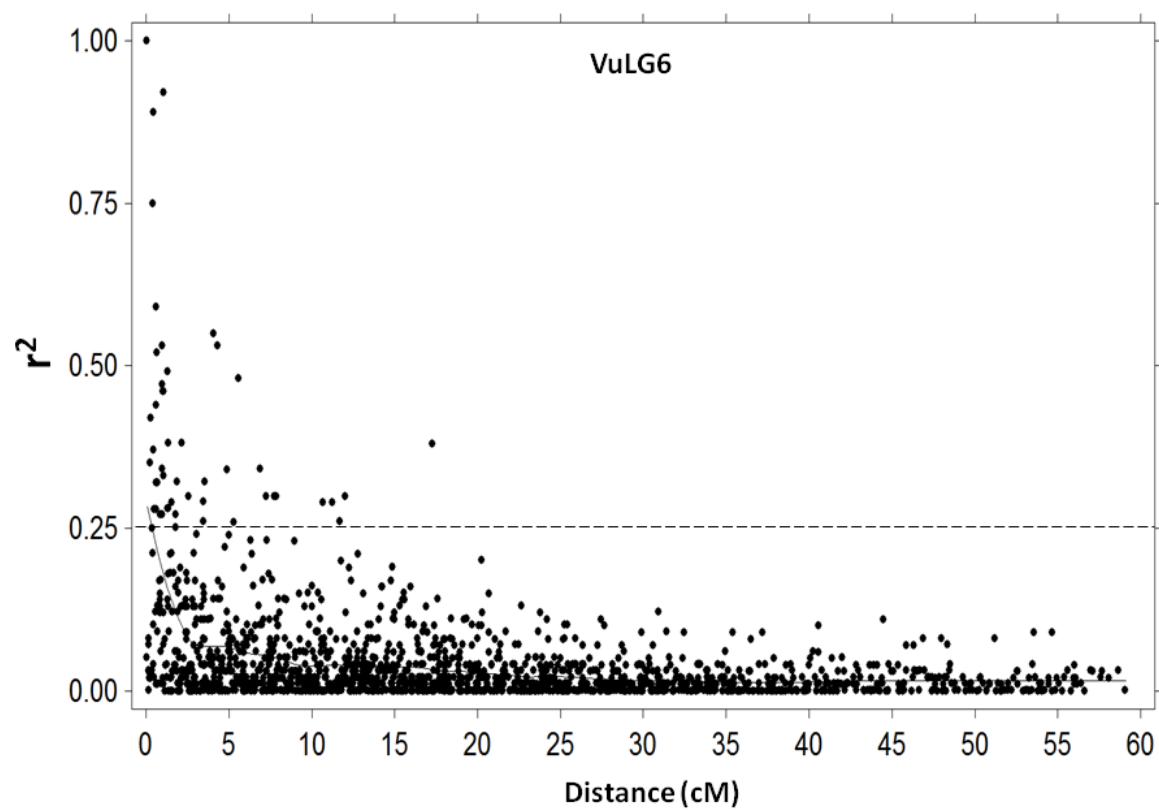

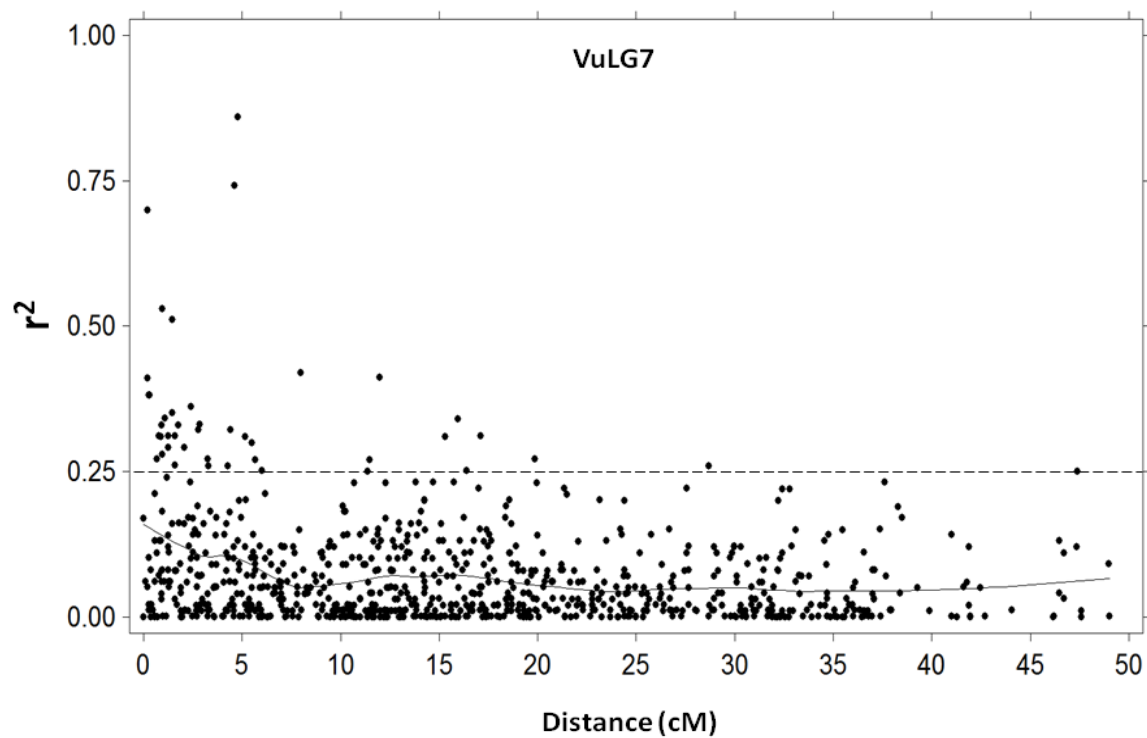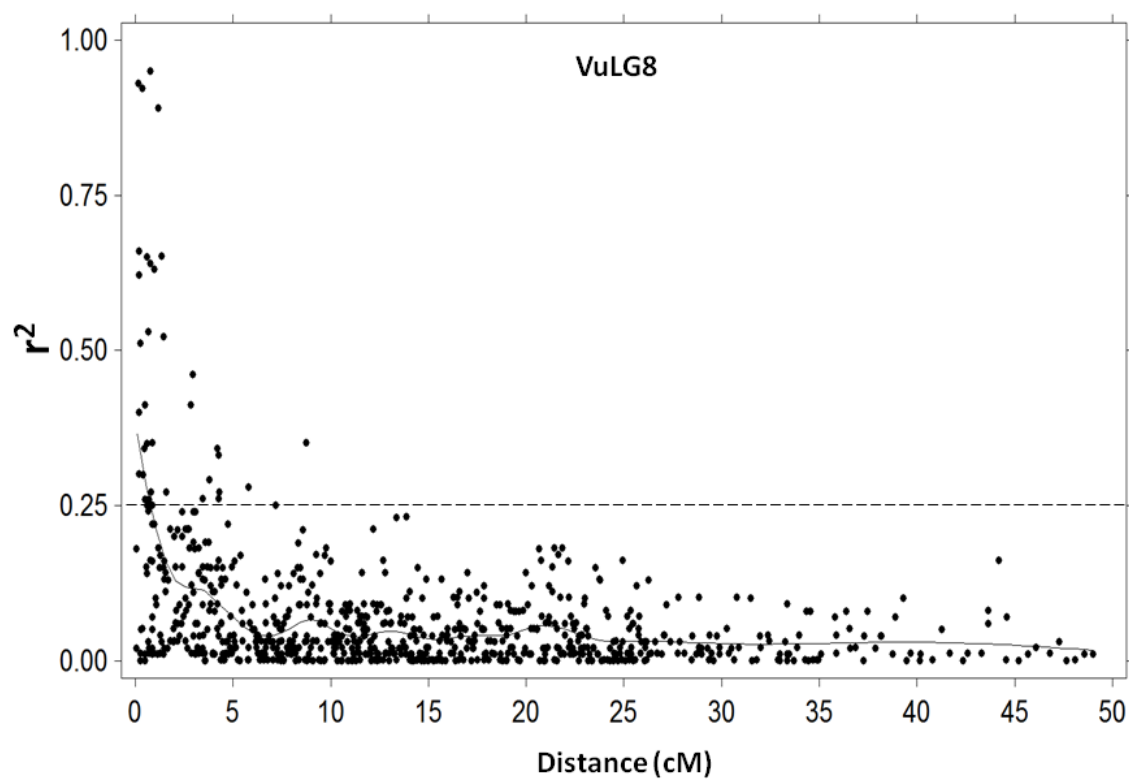

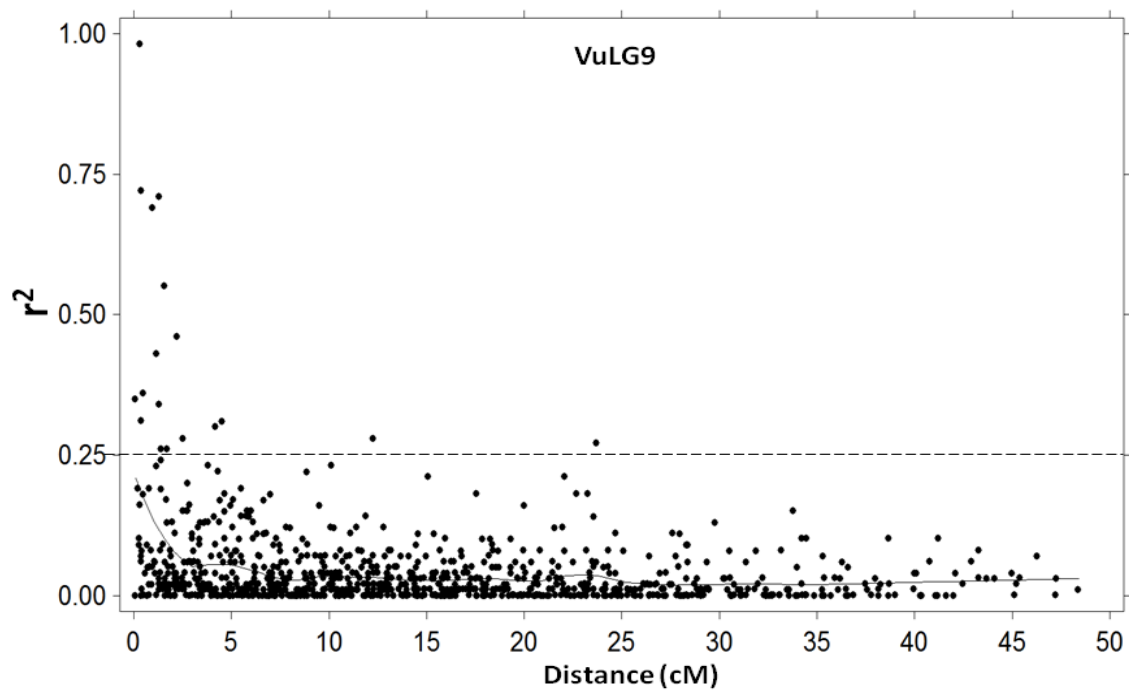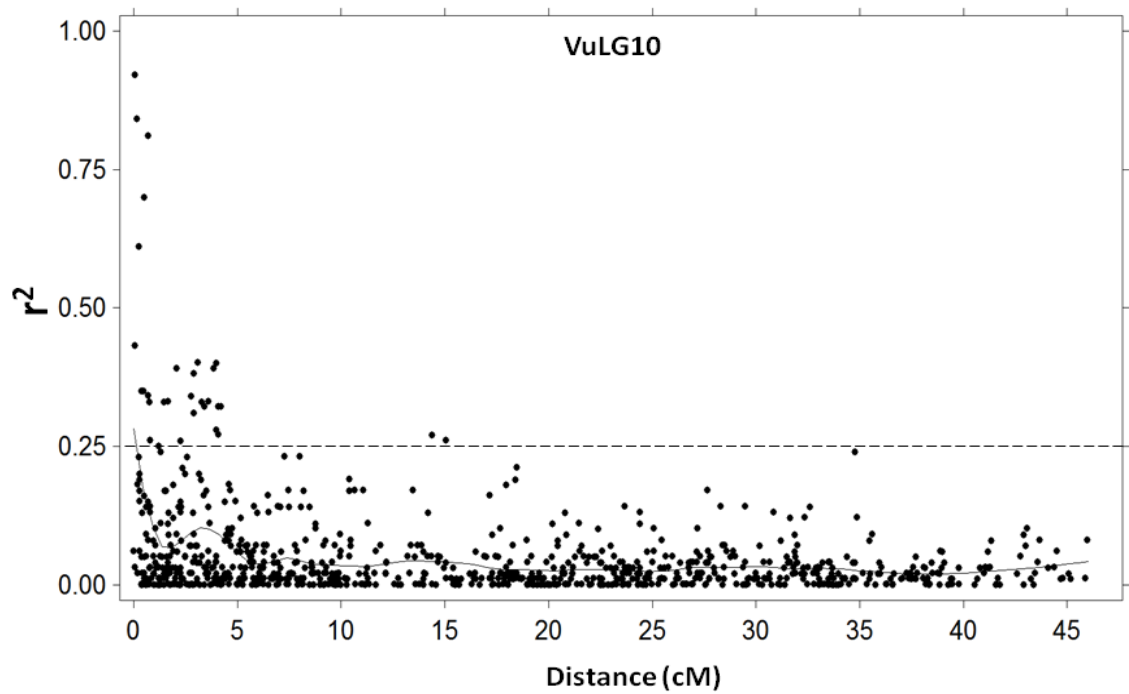

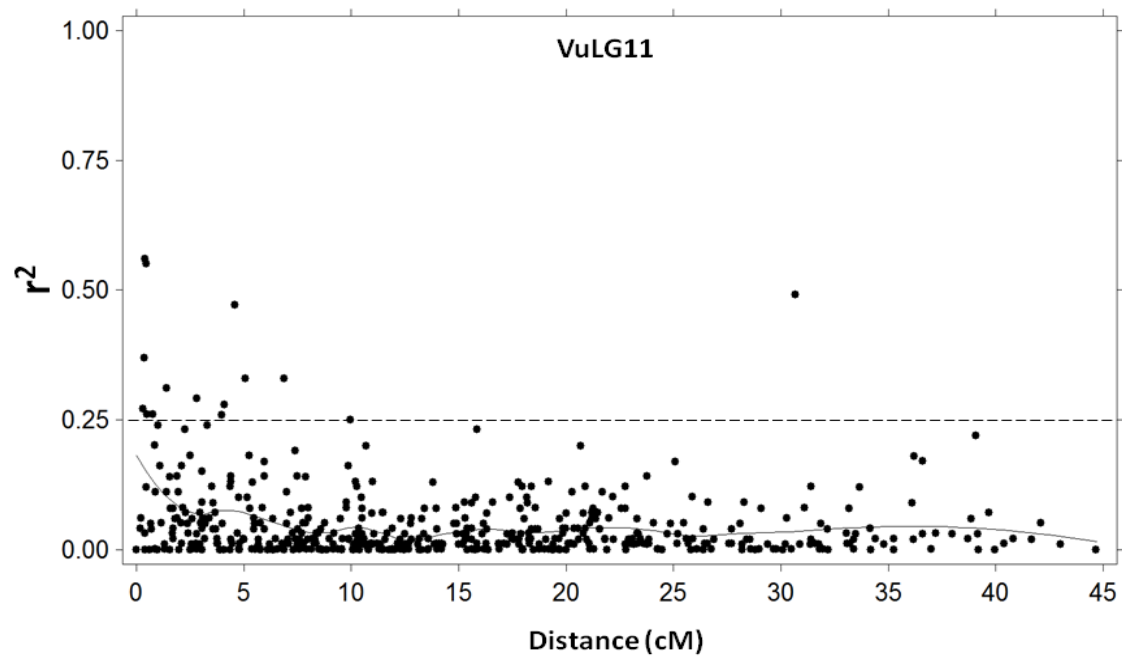

Supplement: Figure S1 — LD decay curves for eleven linkage groups of the cowpea consensus genetic linkage map. (PDF) [file pone.0070041.s001.pdf]
