# Supplementary material for: Genetic Architecture of Delayed Senescence, Biomass, and Grain Yield under Drought Stress in Cowpea
Source: PLoS One. 2013 Jul 30;8(7):e70041. doi: 10.1371/journal.pone.0070041 (PMC3728364; doi:10.1371/journal.pone.0070041)
Supplement: Table S4 — Details of experiments conducted to evaluated cowpea response to drought stress. (DOCX) [file pone.0070041.s006.docx]

| Experiment | Location | Date Planted | Number of Genotypes/RILs evaluated | Replication | Phenotypes evaluated |
| --- | --- | --- | --- | --- | --- |
| BF_2008 | Kamboinse, Burkina Faso | August 29, 2008 | 200 genotypes/49RILs | 3 | Biomass yield, Grain yield, 100-seed weight, PFS^†^ |
| BF_2009A | Pobe-mengao, Burkina Faso | August 1, 2009 | 200 genotypes | 3 | Biomass yield, Grain yield components^‡^ |
| BF_2009B | Pobe-mengao, Burkina Faso | August 13, 2009 | 200 genotypes | 3 | Biomass yield, Grain yield components |
| BF_2009C | Kamboinse, Burkina Faso | August 21, 2009 | 200 genotypes | 3 | Biomass yield, Grain yield components |
| Greenhouse 1 | Riverside, CA, USA | September 25, 2008 | 205 genotypes | 4 | Seedling senescence |
| Greenhouse 2 | Riverside, CA, USA | November 17, 2008 | 205 genotypes | 4 | Seedling senescence |
| Nigeria_2007 | Kano, Nigeria | October 11, 2007 | 339 genotypes | 4 | Biomass yield, Grain yield, Post-flowering senescence |
| Senegal_2008 | Bambey, Senegal | October 29, 2008 | 162 genotypes/51 RILs | 4 | Biomass yield, Grain yield, 100-seed weight, PFS |
| USA_2007A | Coachella, CA, USA | August 16, 2007 | 99 genotypes/57 RILs | 4 | Grain yield |
| USA_ 2007B | Coachella, CA, USA | August 16, 2007 | 99 genotypes/57 RILs | 4 | Grain yield |
| USA_ 2007C | Coachella, CA, USA | August 16, 2007 | 99 genotypes/57 RILs | 4 | Grain yield |
| USA_2008 | Coachella, CA, USA | August 20, 2008 | 201 genotypes/84 RILs | 4 | Grain yield |
| USA_2009 | Coachella, CA, USA | April 15, 2009 | 200 genotypes | 4 | Early vegetative senescence |

Table S4 Details of experiments conducted to evaluated cowpea response to drought stress

^†^PFS = Post-flowering senescence

^‡^ Grain yield components = Pod number per plant, Pod weight per plant, Seed weight per Plant, 100-seed weight , Seed Number per plant, Seed number per pod,
