# Supplementary material for: Genetic Architecture of Delayed Senescence, Biomass, and Grain Yield under Drought Stress in Cowpea
Source: PLoS One. 2013 Jul 30;8(7):e70041. doi: 10.1371/journal.pone.0070041 (PMC3728364; doi:10.1371/journal.pone.0070041)
Supplement: Table S5 — Summary of climatic conditions for field experiments conducted Burkina Faso (BF), Nigeria, Senegal, and the United States of America (USA). (DOCX) [file pone.0070041.s007.docx]

| Experiment | Months | Max. Air Temp (^o^C) | Min. Air Temp (^o^C) | Evapo-transpiration (mm) | Total Precipitation (mm) |
| --- | --- | --- | --- | --- | --- |
|  |  |  |  |  |  |
| BF_2008 | Aug, Sept, Oct | 32.1, 33.1, 36.8 | 22.8, 23.0, 22.5 | 40.0, 50.0, 58.1 | 707.8 |
| BF_2009A&B | Aug, Sept, Oct | 31.8, 34.0, 35.1 | 24.2, 23.5, 28.1 | 58.1, 58.0, 76.1 | 344.0 |
| BF_2009C | Aug, Sept, Oct, | 31.1, 33.6, 37.0 | 22.5, 23.0, 24.2 | 62.0, 50.0, 59.3 | 711.7 |
| Nigeria_2007 | Oct, Nov, Dec, Jan, | 31.0, 33.0, 33.2, 26.4 | 24.3, 23.7, 21.4, 23.2 | 34.1, 62.0, 68.1, 73.3 | 4.0 |
| Senegal_2008 | Oct, Nov, Dec, Jan | 35.3, 37.2, 34.4, 33.4 | 22.3, 18.4, 16.3, 16.7 | 30.3, 60.6, 70.0, 70.5 | 130.0 |
| USA_2007 | Aug, Sept, Oct, Nov, Dec | 39.9, 35.3, 30.4, 27.0, 18.5 | 23.8, 20.2, 14.7, 11.0, 4.4 | 166.7, 141.9, 102.4, 65.1, 49.7 | 0.0 |
| USA_2008 | Aug, Sept, Oct, Nov, Dec | 38.7, 38.6, 32.6, 27.9, 18.7 | 24.0, 22.8, 15.6, 12.5, 5.2 | 158.3, 151.9, 111.3, 70.0, 50.3 | 0.0 |
| USA_2009 | Apr, May, June, Jul | 27.7, 35.6, 33.5, 40.9 | 12.6, 20.0, 19.8, 24.5 | 159.5, 198.9, 176.2, 207.0 | 0.0 |

Table S5 Summary of climatic conditions for field experiments conducted Burkina Faso (BF), Nigeria, Senegal, and the United States of America (USA).
